# Investigation of a New Type I Baeyer–Villiger Monooxygenase from *Amycolatopsis thermoflava* Revealed High Thermodynamic but Limited Kinetic Stability

**DOI:** 10.1002/cbic.201900501

**Published:** 2020-01-09

**Authors:** Hamid R. Mansouri, Marko D. Mihovilovic, Florian Rudroff

**Affiliations:** ^1^ Institute of Applied Synthetic Chemistry TU Wien Getreidemarkt 9 1060 Vienna Austria

**Keywords:** biocatalysis, enzyme catalysis, enzyme stability, in silico analysis, monooxygenases

## Abstract

Baeyer–Villiger monooxygenases (BVMOs) are remarkable biocatalysts, but, due to their low stability, their application in industry is hampered. Thus, there is a high demand to expand on the diversity and increase the stability of this class of enzyme. Starting from a known thermostable BVMO sequence from *Thermocrispum municipale* (*Tm*CHMO), a novel BVMO from *Amycolaptosis thermoflava* (BVMO_Flava_), which was successfully expressed in *Escherichia coli* BL21(DE3), was identified. The activity and stability of the purified enzyme was investigated and the substrate profile for structurally different cyclohexanones and cyclobutanones was assigned. The enzyme showed a lower activity than that of cyclohexanone monooxygenase (CHMO_Acineto_) from *Acinetobacter* sp., as the prototype BVMO, but indicated higher kinetic stability by showing a twofold longer half‐life at 30 °C. The thermodynamic stability, as represented by the melting temperature, resulted in a *T*_m_ value of 53.1 °C for BVMO_Flava_, which was comparable to the *T*_m_ of *Tm*CHMO (Δ*T*_m_=1 °C) and significantly higher than the *T*_m_ value for CHMO_Acineto_ ((Δ*T*_m_=14.6 °C)). A strong deviation between the thermodynamic and kinetic stabilities of BVMO_Flava_ was observed; this might have a major impact on future enzyme discovery for BVMOs and their synthetic applications.

## Graphical abstract

**In silico screening**: A Baeyer–Villiger monooxygenase (BVMO) from a thermophilic origin is identified through an in silico approach. There is a strong deviation between the thermodynamic and kinetic stabilities of the new BVMO, which might have a major impact for future enzyme discovery of BVMOs and their synthetic applications.

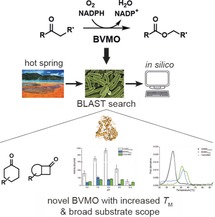

## Introduction

Baeyer–Villiger monooxygenases (BVMOs) were identified, isolated, and characterized in the late 1960s and since then have become highly versatile biocatalysts for the oxidation of ketones and aldehydes into the corresponding esters or lactones (Baeyer–Villiger reaction).^1^ These enzymes utilize molecular oxygen and operate at ambient temperatures and under slightly basic conditions, whereas conventional chemical reactions often require explosive and hazardous oxidants, such as peracids.^2^ Based on the type of cofactor accepted by the enzyme, two different BVMO types can be classified. Type I is tightly bound to flavin adenine dinucleotide (FAD) as a cofactor and uses reduced nicotinamide adenine dinucleotide phosphate (NADPH) as a source of electrons, whereas type II relies on flavin mononucleotide (FMN) and uses reduced nicotinamide adenine dinucleotide (NADH) as an electron donor.^3^ Type I BVMOs catalyze the oxidation of ketones into esters or lactones,^4^, ^5^ with exceptionally high regio‐, chemo‐, and enantioselectivity for the production of fine chemicals or chiral building blocks.2a, 6 Based on these features, many industrial applications^7^ have been suggested, but, due to low operational stability^8^ under given reaction conditions, exploitation on a large scale is still challenging.^9^ Researchers attempted to overcome this limitation by applying different approaches, such as reaction engineering,^10^ protein engineering,^8^, ^11^ and metagenome mining.^12^ For example, Goncalves and co‐workers could increase the kinetic stability of CHMO_Acineto_ 1000‐fold by performing reaction engineering.10a They used a combination of redox cofactors (NADPH and FAD) and natural catalytic antioxidants, such as superoxide dismutase and catalase, to stabilize the enzyme. The only industrial application for BVMOs was published by Bong et al.,^13^ in which the final step of esomeprazole synthesis was catalyzed by a heavily mutated BVMO variant (41 mutations). A different strategy is based on in silico methods by sequence similarity analysis. By exploring metagenomes, it is possible to find new BVMOs, which may show higher stability and a broad substrate acceptance, while avoiding tedious protein engineering. One of the most stable BVMOs, to date, phenylacetone monooxygenase (PAMO) from thermophilic actinomycete *Thermobifida fusca* was found by using this method.12b Recently, genome mining also guided Fraaije and co‐workers to find two other thermostable cyclohexanone monooxygenases (*Tm*CHMO and PockeMO), which were isolated from thermophilic bacteria.12a, 14

Intrigued by the work of Romero et al.,^14^ we aimed to find a novel BVMO with altered thermodynamic stability, but that maintained high activity and substrate acceptance, based on a sequence similarity in silico approach. We envisaged exploiting the sequence space of thermophilic bacteria by using the *Tm*CHMO sequence as a starting point. Among sequences found in the NCBI databank, a new putative BVMO sequence from the thermophilic organism *Amycolatopsis thermoflava*, which was isolated from heat‐treated soil,^15^ was selected. Multiple sequence alignment with different BVMOs^16^, ^17^, ^18^, ^19^ was performed to investigate the Type I BVMO family motifs. The enzyme was cloned and expressed successfully, enzyme activity and stability (kinetic and thermodynamic) were measured, and the substrate profile of this novel BVMO was investigated.

## Result and Discussion

First, we blasted against the NCBI database using *Tm*CHMO as search query; this is one of the most thermostable BVMOs reported, to date. The most similar sequence to that of *Tm*CHMO among thermophilic bacteria was selected and identified as a new putative BVMO from *A. thermoflava* (BVMO_Flava_). The sequence similarity between BVMO_Flava_ and *Tm*CHMO was 83 % and contained the conserved consensus (G/AGxWxxxxF/YPG/MxxxD and FxGxxxHxxxWP/D) of the type I BVMO family. Moreover, both Rossmann‐fold motifs (GxGxxG/A), which are responsible for dinucleotide binding, were identified in BVMO_Flava_. The full alignment is depicted in Figure S1 in the Supporting Information_._

**Figure 1:**
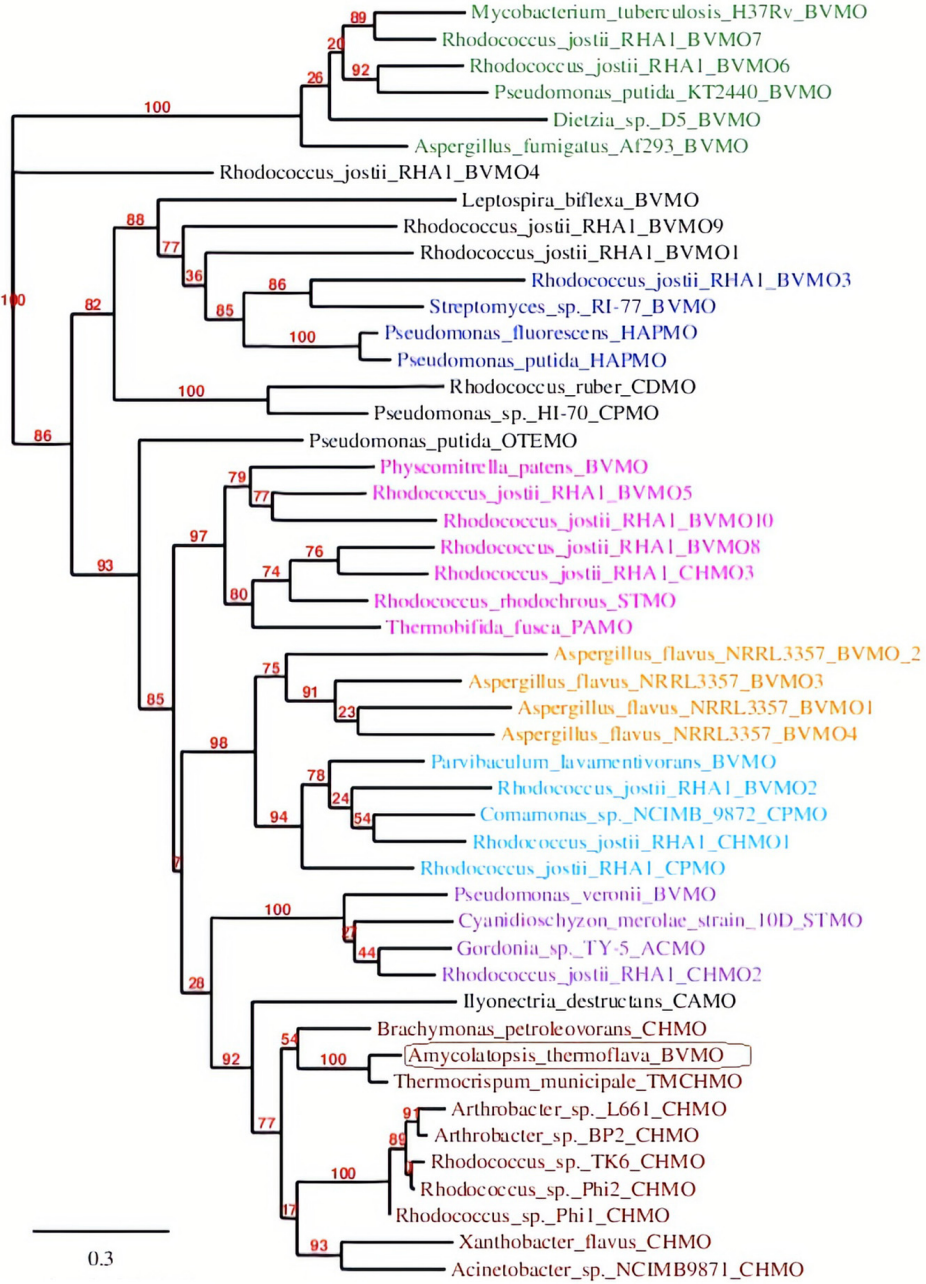
The sequences of recombinantly expressed BVMOs make up the phylogenetic tree, which has been constructed by using PhyML and visualized by Inkscape. Different BVMOs are color‐coded based on the group to which they belong: group 1 (light blue), group 2 (pink), group 3 (maroon), group 4 (blue), group 5 (green), group 6 (orange), and group 7 (violet). BVMO_Flava_ is located in group 3 (red). The accession code of the sequences can be found in Table S1.

Furthermore, we performed a phylogenetic tree analysis with BVMO_Flava_. The phylogenetic tree was constructed by PhyML and visualized by TreeDYN to find the position of BVMO_Flava_ between different groups of BVMOs (Figure 1). The midpoint rooted maximum likelihood phylogram shows the diversity of different BVMOs from groups 1 to 7.^20^ As observed in the maximum likelihood phylogram (Figure 1), the sequence of BVMO_Flava_ is close to the sequence *Tm*CHMO, with a strong bootstrap statistical support of 100 %. The tree also shows that BVMO_Flava_ is placed in the clade of the CHMO family and, in particular, it is a close neighbor of CHMO_Acineto._ This suggests that BVMO_Flava_ displays a similar substrate profile to that of CHMO_Acineto_. A closer look at the structure of BVMO_Flava_ (based on sequence homology towards that of *Tm*CHMO) revealed a high similarity to *Tm*CHMO, whereas for CHMO_Acineto_ small differences, especially in the outer regions and some loops, were observed (Figure S2). This could be an indication that the flexibility of BVMO_Flava_ is hampered, and therefore, the structural stability could be increased.

### Expression and purification

With this novel putative type I BVMO sequence in hand, we ordered the synthetic gene already cloned into a pET22b(+) expression vector with a His‐tag on the C terminus. Subsequently, *Escherichia coli* BL21(DE3) was transformed and protein expression was performed in the presence of isopropyl‐β‐d‐thiogalactopyranoside (IPTG, 50 μm) at 20 °C for 20–22 h. Successful expression was analyzed by means of SDS‐PAGE (Figure S3). A 59.5 kDa band of the purified enzyme was found that belonged to the new BVMO_Flava_. Purification was performed by using a standard HisTrap affinity column.

### Activity and stability measurements

**Figure 2:**
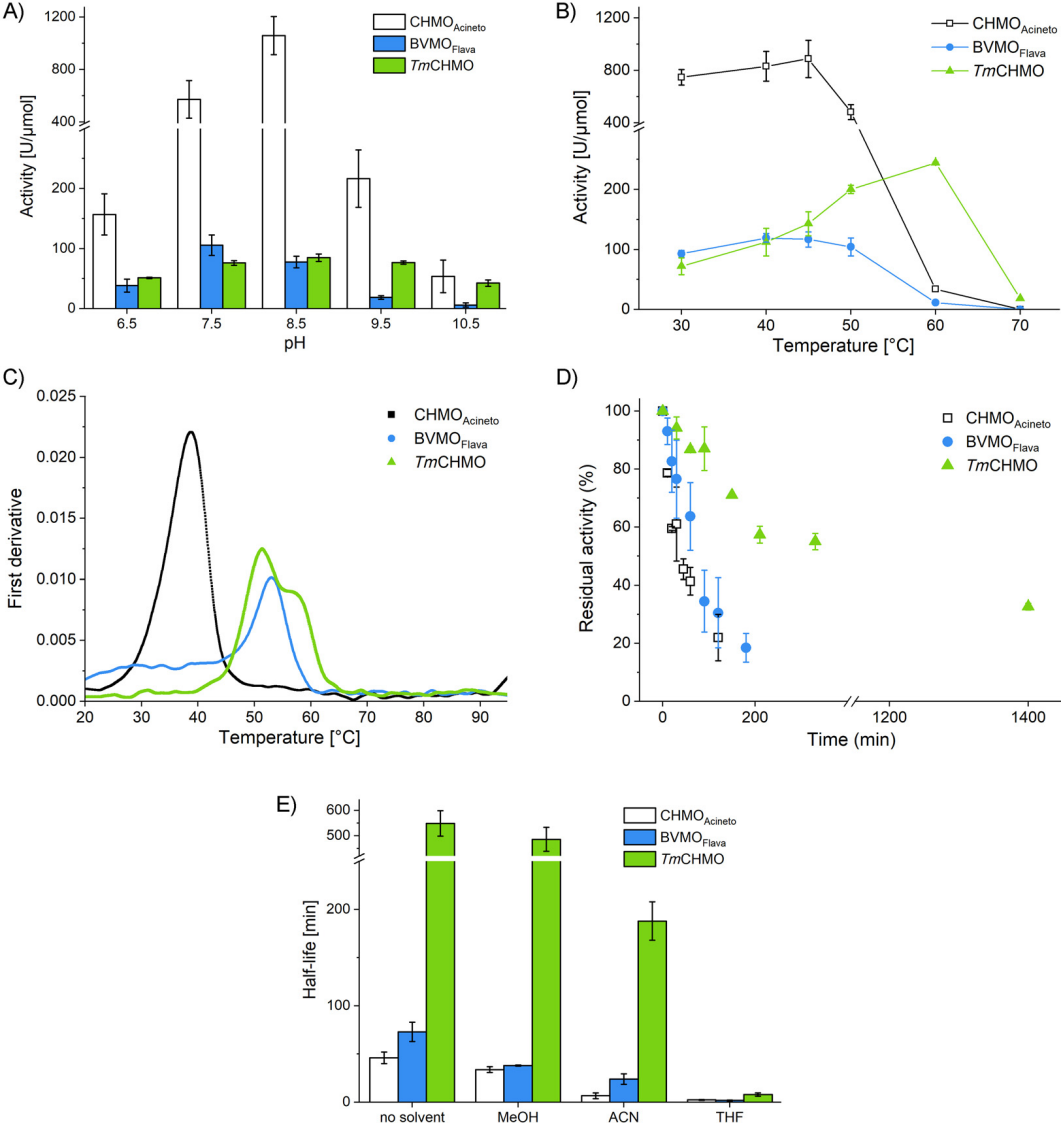
A) Effect of pH on activity at 30 °C in 50 mm Tris**⋅**HCl+10 μm FAD, 0.5 mm cyclohexanone, and 100 μm NADPH. B) Activity measurements at various temperatures from 30 to 70 °C (same conditions as those used for the pH study). C) Melting temperature determination was performed by means of nanodifferential scanning fluorimetry (nanoDSF): 50 mm Tris**⋅**HCl, 10 μm FAD, 2 mg mL^−1^ enzyme. D) Half‐life measurements: incubation at 30 °C, 10 μm enzyme, 50 mm Tris**⋅**HCl, 10 μm FAD, pH 7.5. E) Determination of half‐life in the presence of 5 % cosolvent (same conditions as those used for half‐life measurements). ACN: acetonitrile.

After successful soluble protein expression and purification, we investigated the activity and stability of BVMO_Flava_. First, we had to find a suitable substrate, since the natural one was unknown. Based on the sequence similarity to CHMO_Acineto_, we assumed a comparable substrate profile and tested cyclohexanone as a model compound. Indeed, cyclohexanone was converted into the corresponding ϵ‐caprolactone. The activity was comparable to that of *Tm*CHMO and approximately tenfold lower than that of CHMO_Acineto_ (Figure 2). Kinetic values have been studied for all three enzymes. The *K*_m_ value for cyclohexanone and BVMO_Flava_ was (0.53±0.1) μm, for *Tm*CHMO it was below <1 μm, 14 and for CHMO_Acineto_ it turned out to be one order of magnitude higher ((6.74±2) μm). Next, we determined *k*_cat_, which resulted in the highest value for CHMO_Acineto_ of (15±1.3) s^−1^, relative to those of BVMO_Flava_ ((1.5±0.1) s^− 1^) and *Tm*CHMO (2.0 s^−1^; Table S2). In the following, we investigated the optimum pH, temperature profile, thermodynamic stability, and kinetic stability of BVMO_Flava_, in comparison to those of CHMO_Acineto_ and *Tm*CHMO. The optimum pH for the activity was measured at different pH values, ranging from 7.5 to 10.5, with an interval of 1 pH unit (Figure 2 A). BVMO_Flava_ showed the highest activity at pH 7.5; CHMO_Acineto_ has its optimum at 8.5, whereas *Tm*CHMO was equally active from 7.5 to 9.5 (Figure 2 A). Especially at higher pH values (10.5), *Tm*CHMO outperformed BVMO_Flava_ and CHMO_Acineto_ to maintain 50 % of its initial activity. Next, we determined the optimum temperature for all three enzymes, which turned out to be 45 °C for both BVMO_Flava_ and CHMO_Acineto_, whereas *Tm*CHMO showed the highest activity at 60 °C (Figure 2 B). This result was in contrast to our expectations because the sequence of BVMO_Flava_ originated from a thermophilic organism. A different picture was observed by comparing the thermodynamic stability by recording their melting temperatures (*T*_m_; Figure 2 C). BVMO_Flava_ showed the highest *T*_m_ ((53.1±0.2) °C), whereas *Tm*CHMO and CHMO_Acineto_ showed *T*_m_ values of (52.1±0.6) and (38.5±0.1) °C, respectively (Figure 2 C). This finding is in agreement with the origin of the sequence based on thermostable *Tm*CHMO. Interestingly, *Tm*CHMO showed a second transition midpoint that might indicate an unfolding and deactivation process with two active native states. If the temperature exceeds the second limit, the enzyme goes into the unfolded and deactivated state.

Next, we investigated the kinetic stability (half‐life=*t*_1/2_) of all three BVMOs at 30, 40, and 60 °C (for detailed reaction conditions, see the Experimental Section). At 30 °C, BVMO_Flava_ is approximately twofold ((73±10) min) more stable than that of CHMO_Acineto_ ((46±6) min) and 7.5‐fold less stable than that of *Tm*CHMO ((549±51) min, Figure 2 D). A similar picture was observed after incubation for 1 h at 40 °C. CHMO_Acineto_ showed a *t*_1/2_ of 2.02±0.45 min, whereas BVMO_Flava_ was three times more stable, with a *t*_1/2_ of (6.00±0.80) min. In contrast, *Tm*CHMO still shows more than 60 % of its residual activity. We investigated the *t*_1/2_ of *Tm*CHMO at 60 °C and determined a half‐life of (0.8±0.2) min (Figure S5 A and B). These results also confirmed our previous finding that thermodynamic stability did not necessarily correlate with kinetic stability within BVMO biocatalysts.10a Moreover, we also investigated the stability of all three enzymes in the presence of different organic solvents (5 % *v*/*v*; Figure 2 E). All of them showed a decent stability in the presence of MeOH, whereas 5 % (*v*/*v*) ACN affected CHMO_Acineto_ the most and resulted in almost complete loss of activity. The most destructive cosolvent for the investigated enzymes was THF, which led immediately to the complete deactivation of all three enzymes.

### Substrate profile of BVMO_Flava_

After full biochemical characterization of novel BVMO_Flava_, we elucidated its substrate profile for potential industrial applications and compared it to literature data for CHMO_Acineto_ and *Tm*CHMO. We applied whole‐cell biotransformations under nongrowing conditions and analyzed the performance (conversion and enantiomeric excess (*ee*)) by means of chiral gas chromatography after 24 h of reaction time. Positive control experiments were performed with cyclohexanone. First, Baeyer–Villiger oxidation of different substituted cyclohexanones and cyclobutanones were studied.

The substrate acceptance and enantiopreference of 4‐substituted cyclohexanones **1 a**–**d** are comparable for all three enzymes (Table 1). The only minor exception was found for the bulky substrate **1 c**, which gave full conversion in the presence of BVMO_Flava_ and a very high optical purity (96 % *ee*) of the desired lactone (Table 1). A similar trend was observed for substrates **2** and **3**; all three enzymes showed the same catalytic performance with respect to conversion and enantiopreference.

**Table 1:** Baeyer–Villiger reactions with substituted cyclic ketones.

Substrate	R			Reference reaction			
		BVMO_Flava_		CHMO_Acineto_		*Tm*CHMO	
		Conv [%]^[a]^	*ee* [%]^[b]^	Conv [%]	*ee* [%]	Conv [%]	*ee* [%]^14^
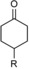	**1 a** R=Me	>99	99 (*S*)	>99	98 (*S*)^27^	>99	99 (*S*)
	**1 b** R=OH	83	8 (*R*)	81	10 (*R*)^28^	89	18 (*R*)
	**1 c** R=*t*Bu	>99	96 (*S*)	17	>98 (*S*)^28^	>99	93 (*S*)
	**1 d** R=Ph	34	89 (−)	30	60 (−)^29^	82	88 (−)
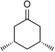	**2**	59	99 (4*S*,6*R*)	85	99 (4*S*,6*R*)^30^	84	99 (4*S*,6*R*)
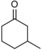	**3**	>99	P/D 41:59^[c]^	>99	P/D 49:51	>99	P/D 49:51
			>99 (−), 96 (−)		99 (−), 99 (−)^31^		99 (−), 99 (−)
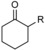	**4 a** R=Ph	40	94 (*R*)	41	98 (*R*)^31^	49	97 (*R*)
	**4 b** R=Bn	42	99 (*R*)	38	96 (*R*)^31^	48	98 (*R*)
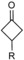	**5 a** R=Ph	10	17 (*R*)	94	62 (*R*)^29^	>99	49 (*R*)
	**5 b** R=Cl‐Ph	16	77 (*S*)	83	81 (*S*)^29^	81	95 (*S*)
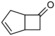	**6**	>99	N/ABN 50:50^[d]^	>99	N/ABN 51:49	>99	N/ABN 50:50
			>99 (−), >99 (−)		95 (−), >99 (−)^31^		>99 (−), >99 (−)
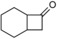	**7**	>99	N/ABN 59:41	>99	N/ABN 65:35	>99	N/ABN 55:45
			72 (−), >99 (−)		60 (−), >95 (−)^31^		79 (−), >98 (−)

[a] Relative conversion (Conv) of substrate to product. [b] Enantiomeric excess (*ee*) of product. [c] The proximal to distal (P/D) ratio of the lactone. [d] Ratio of normal to abnormal (N/ABN) lactone.

The kinetic resolution of **4 a** and **4 b** resulted in the formation of the *R* enantiomer in up to 99 % *ee* after almost 50 % conversion. Next, four different cyclobutanones were tested (**5**–**7**), and again the same conversions and enantioselectivities were obtained. Compounds **5 a** and **5 b** were poorly accepted by CHMO_Flava_: substrate **5 a** gave almost racemic lactone, whereas **5 b** resulted in the desired lactone with 77 % *ee*. For the fused cyclobutanone **6**, full conversion and perfect optical purities for both the normal and abnormal lactone were achieved. The normal lactone is an intermediate in the synthesis of the Corey lactone, which is a building block for prostaglandin synthesis,^21^ and the abnormal product is a starting material of brown algae pheromone synthesis.2a Within our study, we identified a novel type I BVMO based on a sequence similarity search of recently published and thermostable *Tm*CHMO. The BVMO from *A. thermoflava* showed a high amino acid sequence similarity to that of *Tm*CHMO with a molecular weight of 59.5 kDa.

Based on the phylogenetic tree analysis, BVMO_Flava_ belongs to group 3 of type I BVMOs and is located in the same clade as that of CHMO_Acineto_ and *Tm*CHMO. As expected, a similar substrate profile for all three enzymes was determined. Although BVMO_Flava_ originates from a thermophilic organism, the kinetic stability at slightly elevated temperatures dropped from 72 (at 30 °C) to 6 min at 40 °C. In contrast, the thermodynamic stability (*T*_m_ value) was comparable to that of *Tm*CHMO and significantly higher than that of CHMO_Acineto_. The deviation between kinetic and thermodynamic stability is a major problem in the field, since often only *T*_m_ values are published, without any context to the actual operational performance of the new catalyst. Within this study, we would like to emphasize how important it is to determine both stabilities for future comparison and putative industrial applications of BVMOs.

## Experimental Section

**Materials**: All chemicals and reagents were from commercial sources (New England BioLabs, Ipswich, MA, USA; Promega Corp., Madison, WI, USA; Carl Roth, Karlsruhe, Germany; Lab M Ltd., Lancashire, UK; Sigma–Aldrich Corp., St. Louis, MO, USA; Merck KGaA, Darmstadt, Germany; Chem Lab, Zedelgem, Belgium). All substrates used in this study were either available commercially or synthesized in our laboratory. Distilled solvents were used in this study.

**Sequence analysis**: Multiple sequence alignment was performed by means of MUSCLE (multiple sequence comparison by log‐expectation).^22^ The phylogenetic tree was generated by using *phylogeny.fr*.^23^ The homology model was created by using SWISS‐MODEL^24^ and the protein 3D structure was visualized by using Swiss PDB viewer.^25^ Multiple structure alignment was performed by using PyMOL.

**Plasmid construction, microbial strains, and culture media**: An optimized DNA fragment containing the selected BVMO genes from *A. thermoflava* (WP_027929099.1) and *Tm*CHMO (WP 028849141.1) were synthesized and inserted into pET22b(+) by GeneScript with NdeI and NotI restriction sites, respectively. CHMO_Acineto_ was obtained from Gang Chen et al.^26^ The synthesized gene was confirmed by sequencing using T7 and T7term primers. *E. coli* strain BL21(DE3) was transformed by heat‐shock under standard procedures provided by the Neb Transformation Kit. Transformed cells were grown in an incubator operating at 37 °C in lysogeny broth–agar (LB‐agar) medium supplemented with 100 μg mL^−1^ ampicillin.

**Protein expression**: *E. coli* strain BL21(DE3) was used as an expression host for all enzymes in this study (BVMO_Flava_, CHMO_Acineto_, and *Tm*CHMO). LB medium (5 mL) supplemented with ampicillin to a final concentration of 100 μg mL^−1^ was inoculated with *E. coli* BL21(DE3) pET22b(+)_BVMO_Flava_/CHMO_Acineto_/*Tm*CHMO and incubated in an orbital shaker at 37 °C, 200 rpm, overnight. Precultivated bacteria (2 % *v*/*v*) were transferred to a 1 L flask containing LB (250 mL) with the same concentration of ampicillin as that used before. Cells were incubated at 37 °C, 200 rpm, for 2 h to reach an optical density (OD) between 0.6 and 0.8 at *λ*=590 nm. Then IPTG was added to a final concentration of 50 μm and the flask was transferred to 20 °C and incubated for 18–22 h.

**Enzyme purification**: All further steps were performed at 4 °C to protect the enzyme against inactivation. The overnight culture containing expressed recombinant cells were centrifuged at 8000 *g*, 4 °C, for 10 min and cells were collected. Cell pellets were resuspended in 50 mm Tris**⋅**HCl, pH 7.5, containing 100 μm FAD and 100 μm phenylmethylsulfonyl fluoride (PMSF). The crude cell extract was sonicated by using a Bandelin KE76 sonotrode connected to a Bandelin Sonoplus HD 3200 instrument in nine cycles (5 s pulse, 55 s break, amplitude 50 %). Cell debris and aggregates were removed by means of centrifugation (25 000 *g*, 25 min, 4 °C, JA‐17 Beckmann rotor). Supernatant was filtered by using a 0.25 μm filter; equilibrated with 50 mm Tris**⋅**HCl, pH 7.5, 0.5 m NaCl, and 100 μm FAD; and applied on a Ni‐Sepharose column (1 mL, GE Healthcare Bioscience). The unwanted unattached proteins were washed out by using 5 column volumes of 50 mm Tris**⋅**HCl, 0.5 m NaCl, 40 mm imidazole, and 100 μm FAD, at pH 7.5. The elution step was performed by applying 5 column volumes of 50 mm Tris**⋅**HCl, 0.5 m NaCl, at pH 7.5, containing 400 mm imidazole and 100 μm FAD. The eluted enzymes were washed with 50 mm Tris**⋅**HCl and 100 μm FAD, at pH 8, and concentrated in an ultracentrifuge tube with a cutoff of 10 kDa.10a

**Activity and stability measurements**: Enzyme activity was measured by monitoring the decrease in NADPH absorbance at *λ*=340 nm. Standard assays contained the enzyme (0.05 μm), cyclohexanone (0.5 mm), and NADPH (100 μm) in 50 mm Tris**⋅**HCl, adjusted to the desired pH. All measurements were performed at 30 °C.10a The reaction was started immediately after enzyme addition by adding NADPH (4 μL, 25 mm stock solution) to the cuvette (final volume 1 mL). Oxidation of NADPH was followed at 30 °C in a Lambda 35 spectrophotometer (PerkinElmer, Waltham, MA, USA) for 120 s. Stability measurements were performed by incubating 10 μm enzyme at 30 °C in 50 mm Tris**⋅**HCl, 10 μm FAD, pH 7.5. Samples were taken at different time points and added to a cuvette containing 100 μm NADPH and 0.5 mm substrate to test for catalytic activity. The stability in the presence of different cosolvents (MeOH, ACN, and THF), with a final concentration of 5 %, was measured under the same reaction conditions as those described previously. The experimental data were fitted to an exponential decay equation by using Origin Pro software (Origin 9.1 for Windows). The regression data are depicted in the Supporting Information.

**Melting temperature measurements**: The melting temperatures (*T*_m_) of all three enzymes were measured by using a Prometheus NT.48 instrument. The samples were prepared in Tris**⋅**HCl 50 mm, pH 7.5, and 10 μm FAD with a final enzyme concentration of 2 mg mL^−1^ and the samples were run from 20 to 95 °C.

**Biotransformations**: Recombinant protein expression was performed in LB medium, supplemented with ampicillin (100 μg mL^−1^). Enzyme expression was induced by IPTG (final concentration of 50 μm) at 20 °C. Cells were centrifuged (8000 *g*, 4 °C, 10 min) and resuspended and washed in 50 mm phosphate‐buffered saline (PBS) at pH 7.4. After washing, the cells were centrifuged (8000 *g*, 4 °C, 10 min) and resuspended again with the same buffer to reach OD 30. Recombinant expressed cells (1 mL; OD_590_=30) were suspended in PBS (pH 7.4, 50 mm) to a final concentration of 10 mm substrate (methanol as cosolvent (5 % of total volume)). The components of the reaction (1.02 mL in total) were added to a 25 mL flask, and the reaction was performed at 30 °C by shaking (220 rpm) for 24 h.12a The product was extracted with ethyl acetate containing 0.1 mm methyl benzoate as the internal standard for GC analysis. Product analysis was performed by means of GC (Thermo Scientific Trace or Focus GC, Thermo Fisher Scientific) with a chiral/achiral column. Product validation was performed by comparison to known reference biotransformations reported in the literature. Information on columns and methods for the GC experiments is provided in the Supporting Information.

## Conflict of interest

*The authors declare no conflict of interest*.

## Supporting information

As a service to our authors and readers, this journal provides supporting information supplied by the authors. Such materials are peer reviewed and may be re‐organized for online delivery, but are not copy‐edited or typeset. Technical support issues arising from supporting information (other than missing files) should be addressed to the authors.

Supplementary
