# Supplementary material for: Investigation of a New Type I Baeyer–Villiger Monooxygenase from Amycolatopsis thermoflava Revealed High Thermodynamic but Limited Kinetic Stability
Source: Chembiochem. 2020 Jan 9;21(7):971–7. doi: 10.1002/cbic.201900501 (PMC7187199; doi:10.1002/cbic.201900501)
Supplement: Supplementary file 1 — Supplementary [file CBIC-21-971-s001.pdf]

CHEMBIOCHEM

Supporting Information

Investigation of a New Type I Baeyer–Villiger Monooxygenase from *Amycolatopsis thermoflava* Revealed High Thermodynamic but Limited Kinetic Stability

Hamid R. Mansouri, Marko D. Mihovilovic, and Florian Rudroff*[a]

cbic_201900501_sm_miscellaneous_information.pdf

Investigation of a Novel Type I Baeyer-Villiger Monooxygenase from *Amycolatopsis thermoflava* Revealed High Thermodynamic- but Limited Kinetic Stability

Hamid R. Mansouri[1], Marko D. Mihovilovic[1], Florian Rudroff[1*]

[1]Institute of Applied Synthetic Chemistry, TU Wien, Getreidemarkt 9, 1060, Vienna, Austria

Table of Contents

List of sequences used for phylogeny tree 2
Multiple sequence alignment 5
Multiple structure alignment 7
Enzyme purification 8
Stability graphs at 30 °C with regression data 9
Stability measuerement 9
Kinetic measurement 10
GC conditions 11
Baeyer-Viliiger oxidation of substrates 12
References 13

List of sequences used for phylogenetic tree analysis8

Table S1 BVMO sequences used to infer the phylogenetic tree

Enzyme	Accession number	Amino acids identity
BVMO *Leptospira biflexa*	ABZ97795	-
CHMO *Acinetobacter* sp. NCIMB 9871	BAA86293	140/464 (30%)
BVMO *Acinetobacter radioresistens* S13	ADF32068	117/435 (27%)
CHMO *Arthrobacter* sp. L661	ABQ10653	136/472 (29%)
CHMO *Arthrobacter* sp. BP2	AAN37479	139/472 (29%)
CHMO *Brachymonas petroleovorans*	AAR99068	142/488 (29%)
CHMO1 *Brevibacterium* sp. HCU	AAG01289	133/464 (29%)
CHMO2 *Brevibacterium* sp. HCU	AAG01290	123/499 (25%)
CPMO *Comamonas* sp. NCIMB 9872	BAC22652	125/468 (27%)
BVMO3 *Dietzia* sp. D5	AHE80562	100/417 (24%)
BVMO4 *Dietzia* sp. D5	AGY78320	128/489 (26%)
ACMO *Gordonia* sp. TY-5	BAF43791	130/494 (26%)
BVMO1 *Mycobacterium tuberculosis* H37Rv	CAA97398	201/461 (44%)
BVMO2 *Mycobacterium tuberculosis* H37Rv	CAA17436	96/346 (28%)
BVMO3 *Mycobacterium tuberculosis* H37Rv	CAB06212	108/426 (25%)
BVMO4 *Mycobacterium tuberculosis* H37Rv	CAB02175	176/488 (36%)
BVMO5 *Mycobacterium tuberculosis* H37Rv	CAA16134	185/471 (39%)
BVMO6 *Mycobacterium tuberculosis* H37Rv	CAA16141	120/426 (28%)
BVMO *Oceanicola batsensis* HTCC2597	A3U3H1	129/486 (27%)
BVMO *Parvibaculum lavamentivorans* NCIMB13966	A7HU16	136/497 (27%)
CHMO *Polaromonas* sp. JS666	YP_552312	146/492 (30%)
CPDMO *Pseudomonas* sp. HI-70	BAE93346	130/450 (29%)
CHMO *Pseudomonas aeruginosa* PAO1	AAG04927	169/495 (34%)
BVMO *Pseudomonas fluorescens* DSM50106	AAC36351	172/481 (36%)
HAPMO *Pseudomonas fluorescens* ACB	AAK54073	163/504 (32%)
HAPMO *Pseudomonas putida* JD1	ACJ37423	157/477 (33%)
BVMO *Pseudomonas putida* KT2440	AAN68413	113/420 (27%)
OTEMO *Pseudomonas putida*	AEZ35248	146/504 (29%)
BVMO *Pseudomonas veronii* MEK700	ABI15711	117/442 (26%)
CHMO *Rhodococcus* sp. HI-31	BAH56677	142/474 (30%)
CHMO *Rhodococcus* sp. TK6	AAR27824	143/474 (30%)
CHMO *Rhodococcus* sp. Phi2	AAN37491	141/474 (30%)

CHMO *Rhodococcus* sp. Phi1	AAN37494	139/462 (30%)
BVMO1 *Rhodococcus jostii* RHA1	ABG98452	128/495 (26%)
BVMO2 *Rhodococcus jostii* RHA1	ABG96095	168/464 (36%)
BVMO3 *Rhodococcus jostii* RHA1	ABG95050	135/506 (27%)
BVMO4 *Rhodococcus jostii* RHA1	ABG94866	120/468 (26%)
BVMO5 *Rhodococcus jostii* RHA1	ABG93916	162/481 (34%)
BVMO6 *Rhodococcus jostii* RHA1	ABG93685	164/484 (34%)
BVMO7 *Rhodococcus jostii* RHA1	ABG97785	183/488 (38%)
BVMO9 *Rhodococcus jostii* RHA1	ABH00079	139/491 (28%)
BVMO10 *Rhodococcus jostii* RHA1	ABH00083	148/504 (29%)
BVMO11 *Rhodococcus jostii* RHA1	ABG98471	128/477 (27%)
BVMO12 *Rhodococcus jostii* RHA1	ABG98876	167/457 (37%)
BVMO13 *Rhodococcus jostii* RHA1	ABG95573	102/374 (27%)
BVMO14 *Rhodococcus jostii* RHA1	ABG95240	121/464 (26%)
BVMO15 *Rhodococcus jostii* RHA1	ABG94297	130/488 (27%)
BVMO16 *Rhodococcus jostii* RHA1	ABG94724	114/430 (27%)
BVMO17 *Rhodococcus jostii* RHA1	ABG97009	191/491 (39%)
BVMO18 *Rhodococcus jostii* RHA1	ABG97176	150/499 (30%)
BVMO19 *Rhodococcus jostii* RHA1	ABG97302	166/483 (34%)
BVMO20 *Rhodococcus jostii* RHA1	ABG99184	123/492 (25%)
BVMO21 *Rhodococcus jostii* RHA1	ABH00380	130/488 (27%)
BVMO24 *Rhodococcus jostii* RHA1	ABG97104	145/489 (30%)
STMO *Rhodococcus rhodochrous* IFO 3338	BAA24454	146/497 (29%)
CDMO *Rhodococcus ruber* SC1	AAL14233	136/487 (28%)
BVMO PntE *Streptomyces arenae*	ADO85575	132/514 (26%)
BVMO PtlE *Streptomyces avermitilis* MA-4680	BAC70705	122/447 (27%)
BVMO1 *Streptomyces coelicolor* A3(2)	CAB55657	129/416 (31%)
BVMO2 *Streptomyces coelicolor* A3(2)	CAB59668	178/485 (37%)
BVMO PenE *Streptomyces exfoliatus*	ADO85591	125/513 (24%)
BVMO (InfQ) *Streptomyces* sp. RI-77	BAU98044.1	161/501 (32%)
PAMO *Thermobifida fusca*	Q47PU3	144/527 (27%)
CHMO *Xanthobacter* sp. ZL5	CAD10801	129/442 (29%)
BVMO *Aspergillus clavatus* NRRL1	XP_001270542	149/527 (28%)
BVMO210 *Aspergillus flavus* NRRL3357	XP_002375343	123/466 (26%)
BVMO456 *Aspergillus flavus* NRRL3357	XP_002375466	115/490 (23%)
BVMO619 *Aspergillus flavus* NRRL3357	XP_002383043	133/492 (27%)
BVMO838 *Aspergillus flavus* NRRL3357	XP_002375657	130/512 (25%)
BVMO1 *Aspergillus fumigatus* Af293	XP_747160	136/483 (28%)
BVMO2 *Aspergillus fumigatus* Af293	XP_746949	98/397 (25%)
BVMO3 *Aspergillus fumigatus* Af293	XP_755274	135/489 (28%)
BVMO *Cyanidioschyzon merolae* 10D	BAM80902	148/543 (27%)
CAMO *Cylindrocarpon radicicola* ATCC 11011	AET80001	138/483 (29%)
BVMO *Physcomitrella patens*	XP_001758613	154/532 (29%)

Multiple sequence alignment

```
BVMOflava MS----------------------------------------------------------
TMCHMO    MS----------------------------------------------------------
CHMO      MS----------------------------------------------------------
PAMO      MA----GQTTVD------------------------------------------------
CPMO      MSKVTPQQLSMN------------------------------------------------
CDMO      MT------TSID------------------------------------------------
HAPMO     MSAFNTTLPSLDYDDDTLREHLQGADIPTLLLTVAHLTGDLQILKPNWKPSIAMGVARSG
          *:
BVMOflava ------------------------------------------------------------
TMCHMO    ------------------------------------------------------------
CHMO      ------------------------------------------------------------
PAMO      ------------------------------------------------------------
CPMO      ------------------------------------------------------------
CDMO      ---------REALRRKYAEERDKRI----RPDGNDQYIRLDHVDGWSHDPYMPI------
HAPMO     MDLETEAQVREFCLQRLIDFRDSGQPAPGRPTSDQLHILGTWLMGPVIEPYLPLIAEEAV

BVMOflava TTRTP---------------DVDAIVIGAGFGGIYMLHKLRNELGL-SVTAFEKGGGVGG
TMCHMO    TTQTP---------------DLDAIVIGAGFGGIYMLHKLRNDLGL-SVRVFEKGGGVGG
CHMO      --QKM---------------DFDAIVIGGGFGGLYAVKKLRDELEL-KVQAFDKATDVAG
PAMO      SRRQP-------------PEEVDVLVVGAGFSGLYALYRLR-ELGR-SVHVIETAGDVGG
CPMO      NSVDD---------------TLDVLLIGAGFTGLYQLHHLR-KLGF-KVHLVDAGADVGG
CDMO      TPREP------------KLDHVTFAFIGGGFSGLVTAARLR-ESGVESVRIIDKAGDFGG
HAPMO     TAEEDLRAPRWHKDHVASGRDFKVVIIGAGESGMIAALRFK-QAGV-PFVIYEKGNDVGG
                                 .   .:*.*  *:    ::: .     .   : . ...*

BVMOflava TWYFNRYPGAKSDTEGFVYRYSFDKDLLREWNWTTRYLEQADVLAYLEHVVERFDLGRDI
TMCHMO    TWYWNKYPGAKSDTEGFVYRYSFDKELLREYDWTTRYLDQPDVLAYLEHVVERYDLARDI
CHMO      TWYWNRYPGALTDTETHLYCYSWDKELLQSLEIKKKYVQGPDVRKYLQQVAEKHDLKKSY
PAMO      VWYWNRYPGARCDIESIEYCYSFSEEVLQEWNWTERYASQPEILRYINFVADKFDLRSGI
CPMO      IWHWNCYPGARVDTHCQIYQYSM-PELWGEFNWKELFPNWAQMREYFYFVDKKLELSKDI
CDMO      VWYWNRYPGAMCDTAAMVYMPLLEET---GYMPTEKYAHGPEILEHCQRIGKHYDLYDDA
HAPMO     TWRENTYPGCRVDINSFWYSFSFARGI-----WDDCFAPAPQVFAYMQAVAREHGLYEHI
           *  * ***.  *     *                 :   .::  :   :  .  *

BVMOflava RLNTEVTGAVFDEESDLWTV---TTATGETTTARYLVNALGLLARSNIPDIPGRDGFAGR
TMCHMO    QLNTEVTDAIFDEETELWRV---TTAGGETLTARFLVTALGLLSRSNIPDIPGRDSFAGR
CHMO      QFNTAVQSAHYNEADALWEV---TTEYGDKYTARFLITALGLLSAPNLPNIKGINQFKGE
PAMO      TFHTTVTAAAFDEATNTWTV---DTNHGDRIRARYLIMASGQLSVPQLPNFPGLKDFAGN
CPMO      SFNTRVQSAVFDEQRREWTV---RSLGHQPIRAKFVIANLGFGASPSTPKVEGIEKFKGE
CDMO      LFHTEVTDLVWQEHDQRWRI---STNRGDHFTAQFVGMGTGPLHVAQLPGIPGIESFRGK
HAPMO     RFNTEVSDAHWDESTQRWQLLYRDSEGQTQVDSNVVVFAVGQLNRPMIPAIPGIETFKGP
           ::* *    ::*    * :    :       :. :    *    .  * . * . * *

BVMOflava LVHTNAWPDD----------LD-ITGKRVGVIGTGSTGTQFIIAAAKTASHLTVFQRSPQ
TMCHMO    LVHTNAWPED----------LD-ITGKRVGVIGTGSTGTQFIVAAAKMAEQLTVFQRTPQ
CHMO      LHHTSRWPDD----------VS-FEGKRVGVIGTGSTGVQVITAVAPLAKHLTVFQRSAQ
PAMO      LYHTGNWPHE---------PVD-FSGQRVGVIGTGSSGIQVSPQIAKQAAELFVFQRTPH
CPMO      WYHTALWPQE---------GVD-MAGKRVAIIGTGSSGVQVAQEAALNAKQVTVFQRTPN
CDMO      SFHTSRWDYDYTGGDALGAPMDKLADKRVAVIGTGATAVQCVPELAKYCRELYVVQRTPS
```

```
HAPMO     MFHSAQWDHD----------VD-WSGKRVGVIGTGASATQFIPQLAQTAAELKVFARTTN
            *:  *  :          :.   .:**.:****::. *     *  . .: *. *:.
BVMOflava YCVPSGNGPVDQTEVDRTK-------------ENFDAIWDQVRNSVVAFGFEESGVEAMS
TMCHMO    YCVPSGNGPMDPDEVARIK-------------QNFDSIWDQVRSSTVAFGFEESTVEAMS
CHMO      YSVPIGNDPLSEEDVKKIK-------------DNYDKIWDGVWNSALAFGLNESTVPAMS
PAMO      FAVPARNAPLDPEFLADLK-------------KRYAEFREESRNTPGGTHRYQGPKSALE
CPMO      LALPMHQRQLSADDNNRMR-------------PDMPAAFERRGKCFAGFDFDFVPKNATE
CDMO      AVDERGNHPIDEKWFAQIATPGWQKRWL----DSFTAIWDGVLTDPSELAI---------
HAPMO     WLLPTPD--LHEKISDSCKW-------LLAHVPHYSLWYRVAMAMPQSVGFLE-------
                :  :

BVMOflava VSEEERRKVFQEAWDKGNGFRFMFGTFCDIATN-----------PEANAAAAAFIRAKIA
TMCHMO    VSESERQRVFQQAWDKGNGFRFMFGTFCDIATN-----------PEANAAAAAFIRSKIA
CHMO      VSAEERKAVFEKAWQTGGGFRFMFETFGDIATN-----------MEANIEAQNFIKGKIA
PAMO      VSDEELVETLERYWQEGG--PDILAAYRDILRD-----------RDANERVAEFIRNKIR
CPMO      VSDTERNEILEELWNTGG-FRYWLANFQDYLFD-----------DKANDYVYEFWRDKVR
CDMO      ----EHEDLVQDGWTALG--QRMRAAVGSVPIEQYSPENVQRALEEADDEQMERIRARVD
HAPMO     ----------------------------DVMVDVGYPP-TELAVSARNDRLRQDISAWME
                                      .   :              :          :

BVMOflava -EIVDDPETARKLTPT----DLYAKRPLCNEG-YYETYNRDNVELVSIKENPIREITPAG
TMCHMO    -EIVKDPETARKLTPT----DLYAKRPLCNEG-YYETYNRDNVSLVSLKETPIEEIVPQG
CHMO      -EIVKDPAIAQKLMPQ----DLYAKRPLCDSG-YYNTFNRDNVRLEDVKANPIVEITENG
PAMO      -NTVRDPEVAERLVPK--GYPFGTKRLILEID-YYEMFNRDNVHLVDTLSAPIETITPRG
CPMO      -ARIKDPKVAEKLAPMKKPHPYGTKRPSLEQW-YYEIFNQSNVKLVDVNETPIQRISETG
CDMO      -EIVTDPATAAQLKAW---FRQMCKRPCFHDD-YLPAFNRPNTHLVDTGGKGVERITENG
HAPMO     PQFADRPDLREVLIPD---SPVGGKRIVRDNGTWISTLKRDNVSMI---RQPIEVITPKG
                *     * .         **   .   :    :: *. :       :  *   *

BVMOflava VRTADGIEHPLDVLVFATGFDAVDGNYR---AMDLRGRGGRHISEHWT-GGPTSYLGVST
TMCHMO    VRTSDGVVHELDVLVFATGFDAVDGNYR---AMNLRGRDGRHINEHWT-EGPTSYLGVTK
CHMO      VKLENGDFVELDMLICATGFDAVDGNYV---RMDIQGKNGLAMKDYWK-EGPSSYMGVTV
PAMO      VRTSER-EYELDSLVLATGFDALTGALF---KIDIRGVGNVALKEKWA-AGPRTYLGLST
CPMO      IVTQEG-ETEFDLIVFATGFDAVTGGLT---SIDFRNNEGQSFKDVWS-DGIRTQLGVAT
CDMO      VVVA-GVEYEVDCIVYASGFEFLGTGYTDRAGFDPTGRDGVKLSEHWA-QGTRTLHGMHT
HAPMO     ICCVDGTEHEFDLIVYGTGFHA-SKFLM---PINVTGRDGVALHDVWKGDDARAYLGMTV
          :         .* :: .:**.           ::  .  .  : : *   .  :  *:

BVMOflava AGFPNMFMILGPNGPF---TNLPPSIETQVDWIGELIRHAERTGVRTVEPTAAAEEAWTA
TMCHMO    AGFPNMFMILGPNGPF---TNLPPSIEAQVEWISDLIDKATREGLTTVEPTADAEREWTE
CHMO      NNYPNMFMVLGPNGPF---TNLPPSIESQVEWISDTIQYTVENNVESIEATKEAEEQWTQ
PAMO      AGFPNLFFIAGPGSPS-ALSNMLVSIEQHVEWVTDHIAYMFKNGLTRSEAVLEKEDEWVE
CPMO      AGFPNLLFGYGPQSPA-GFCNGPSSAEYQGDLLIELMNHLRKNDITRIEAQPAAQEAWRK
CDMO      YGFPNLFVLQLMQGAA-LGSNIPHNFVEAARVVAAIVDHVLSTGTSSVETTKEAEQAWVQ
HAPMO     PQFPNMFCMYGPNTGLVVYSTVIQFSEMTASYIVDAVRLLLEGGHQSMEVKTPVFESYNQ
            :**::             .           :   :      .    *        :

BVMOflava TC--AEIADMTLFPKAD-SWIFGANIPG--KRNAVMFYLAGLGAYRAKLRE-VADAGYT-
TMCHMO    TC--AEIANMTLFPKAD-SWIFGANIPG--KRHAVMFYLGGLGNYRRQLAD-VADGGYR-
CHMO      TC--ANIAEMTLFPKAQ-SWIFGANIPG--KKNTVYFYLGGLKEYRSALAN-CKNHAYE-
PAMO      HV--NEIADETLYPMTA-SWYTGANVPG--KPRVFMLYVGGFHRYRQICDE-VAAKGYE-
```

```
CPMO      LI--ADFWDSSLFPRAK-SWYQGNNIPG--KKVESLNFPLGLPTYIAKFKE-SAEQGYA-
CDMO      LL--LDHGRPLGNPECTPGYYNNEGKPAELKDRLNVGYPAGSAAFFRMMDHWLAAGSFD-
HAPMO     RVDEGNALRAWGFSKVN-SWYKNS------KGRVTQNFPFTAVEFWQRTHS-VEPTDYQL
               :       .   .:   .       *      :      :            :

BVMOflava GFELTRENAT----AAV
TMCHMO    GFQLRGERAQ----AVA
CHMO      GFDIQLQRSDIKQPANA
PAMO      GFVLT------------
CPMO      GFSLS-----------K
CDMO      GLTFR------------
HAPMO     G----------------
          *
```

Figure S1. Multiple sequence alignment analysis of $BVMO_{Flava}$ with known BVMOs. 4-hydroxyacetophenone monooxygenase (HAPMO)[1] [*Pseudomonas fluorescens*] (Q93TJ5.1), cyclododecanone monooxygenase (CDMO)[2] [*Rhodococcus ruber*] (AAL14233.1); cyclopentanone 1,2-monooxygenase (CPMO)[3] [*Acidovorax sp. SCN 65-28*] (ODS80058.1); phenylacetone monooxygenase (PAMO)[4] [*Thermobifida fusca] (*WP_011291921.1); cyclohexanone 1,2-monooxygenase (CHMO)[5] [*Acinetobacter sp. NCIMB9871*] ,(BAA86293.1); NAD(P)/FAD-dependent oxidoreductase (TmCHMO)[6] [*Thermocrispum municipale*] (WP_028849141); NAD(P)/FAD-dependent oxidoreductase ($BVMO_{Flava)}$ [*Amycolatopsis thermoflava*] (WP_027929099.1). Rossmann-fold motifs (GxGxxG/A) and consensus sequences of Type I BVMOs (G/AGxWxxxxF/YPG/MxxxD and FxGxxxHxxxWP/D) are highlighted. Multiple sequenc alignment was performed by MAFFT V.7.[7]

Multiple structure alignment

The structures were visualized by PyMOL (Fig. S2). There is a high similarity of TmCHMO (grey) and $BVMO_{Flava}$ (green). This high similarity, increases the possibility of $BVMO_{Flava}$ for being a thermostable enzyme. The structural comparison of $CHMO_{Acineto}$ (pink) and $BVMO_{Flava}$ (green) shows several differences, which are pointed out by the red circles. The nonoverlapping regions can especially be seen on the outer region of the protein and in the loops, which are responsible for the flexibility of the enzyme.

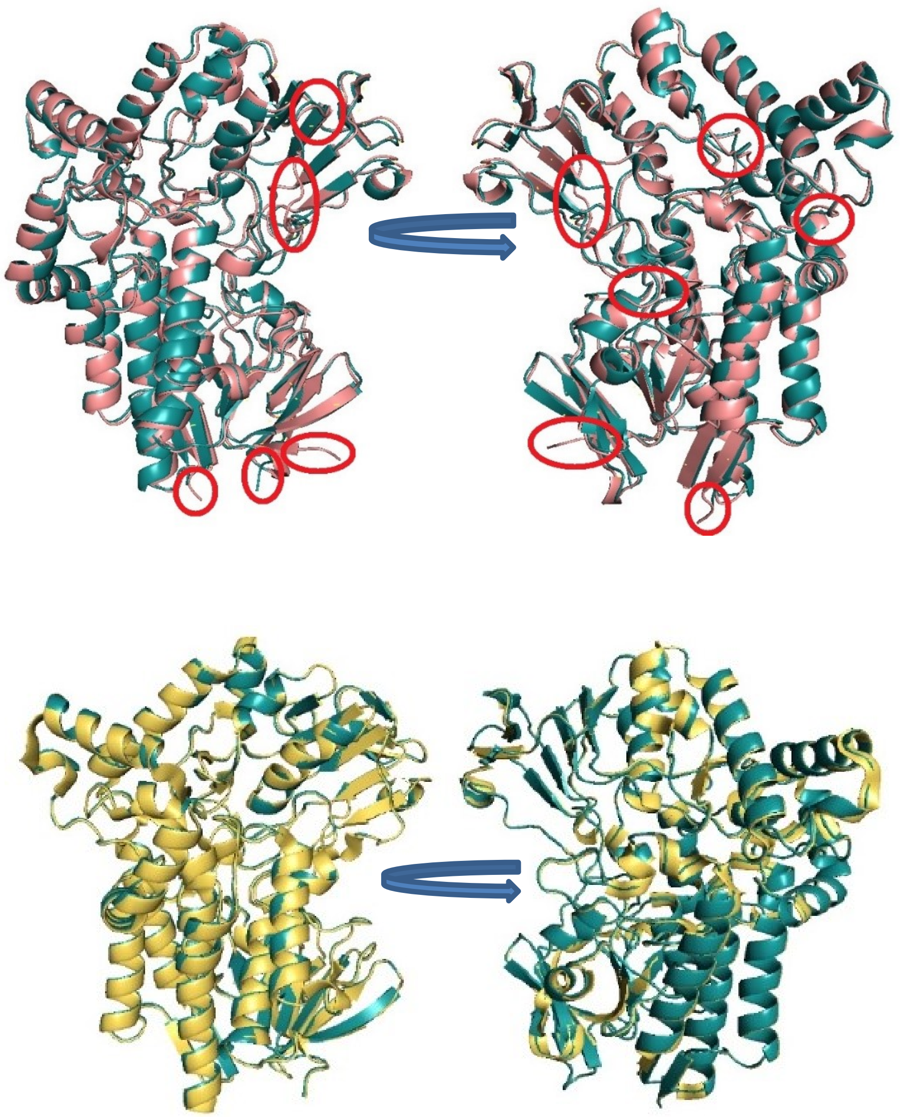

Figure S2 Multiple structure alignment of BVMO$_{\text{Flava}}$ (green), TmCHMO (gold) and CHMO$_{\text{Acineto}}$ (pink). The non-matching regions are highlighted (red oval shape) for CHMO$_{\text{Acineto}}$ and BVMO$_{\text{Flava.}}$

Enzyme purification

Enzyme purification has been performed by His-Trap affinity column (see material and methods of the main manuscript). The pure fractions were concentrated using a ultracentrifugal tube with 10 kDa cut-off. As it can be seen in Figure S3 lane 3, a band for the 59.5 kDa protein was obtained, which belongs to BVMO$_{\text{Flava}}$.

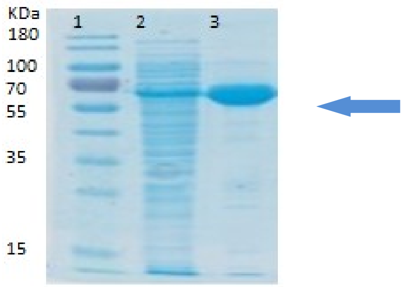

Figure S3 SDS-PAGE analysis of $BVMO_{Flava}$:, lane (1) belongs to standard protein marker, lane (2): cell free extract solution and lane (3) displayed concentrated and purified enzyme.

Stability graphs at 30 °C with regression data

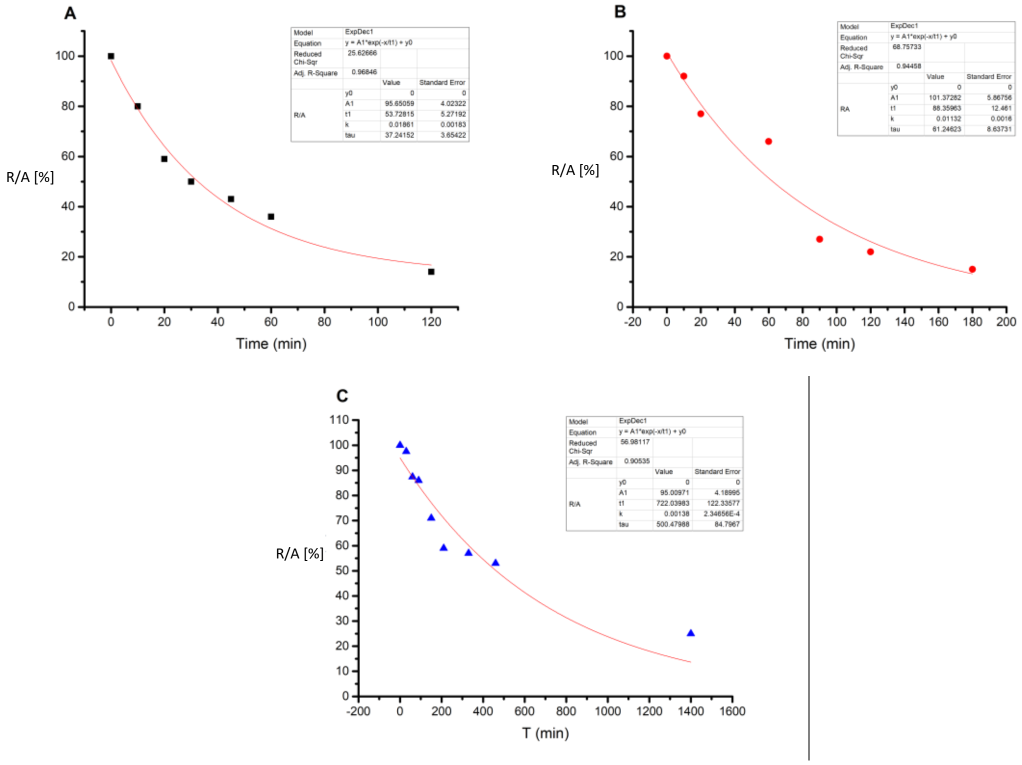

Figure S4 Stability measurement at 30 °C for all three enzymes A) $CHMO_{Acineto}$ B) $BVMO_{Flava}$ c) TmCHMO

Stability measurement

Stability measurement at 40 °C and 60 °C was performed in accordance to the procedure described in the main text.[8]

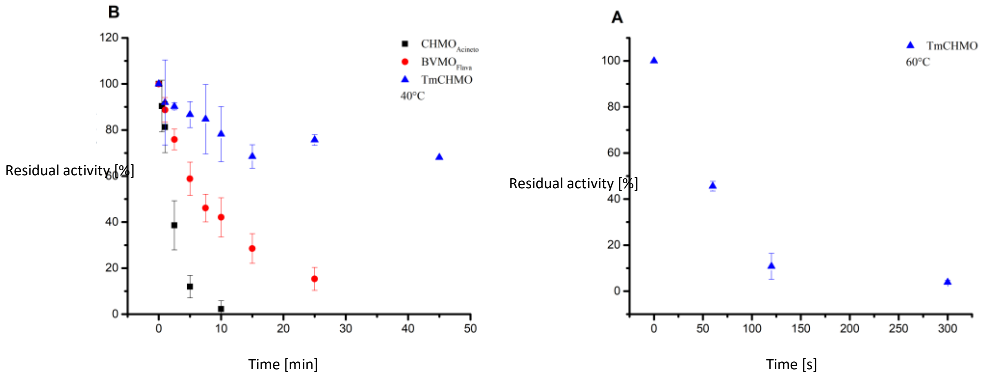

Figure S5 A) Stability measurement at 40 °C B) stability of TmCHMO at 60 °C

Kinetic measurement

In order to measure the catalytic constants (K_m and k_{cat}), reactions were started by mixing the enzyme solution (0.05 µM) with pre-warmed solutions of TrisHCl 50 mM (30 °C) containing NADPH (100 µM) and variable concentrations of cyclohexanone as it can be seen in figure S6.[8]

Table S2 K_m and k_{cat} value measurement

	K_m	StDEv	V_{max}	StDEv	k_{cat}	StDEv	k_{cat}/K_m
Cyclohexanone	[µM]		[U/µmol]		[s^{-1}]		[mM^{-1} s^{-1}]
$CHMO_{Acineto}$	6.74	2	887	45	15.0	1.3	2220
$BVMO_{Flava}$	0.53	0.1	97	4	1.5	0.1	2932
TmCHMO[6]	<1	-	-	-	2	-	>2000

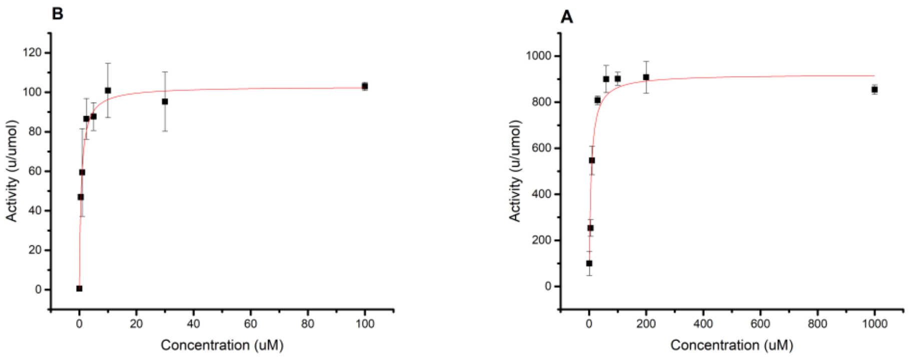

Figure S6 A) kinetic measurement of $CHMO_{Acineto}$ B) kinetic measurement of $BVMO_{Flava}$

GC conditions

Table S3 GC methods

Substrate	Condition	Retention time
1a	80 °C (2 min), 5 °C/min, 160 °C (1 min), 10 °C/min, 220 °C (8 min). Helium: 2 ml/min. Column: BGB173, 30 m x 0.25 mm ID, 0.25 µm.	1a: 6.08 min<br>(S)-2a: 14.33 min<br>(R)-2a : 14.75 min
1b	80 °C (2 min); 2 °C/min, 220 °C (8 min), Helium: 2 ml/min. Column: BGB175, 30 m x 0.25 mm ID, 0.25 µm.	1b: 34.8<br>(S)-2b: 50.997<br>(R)-2b: 51.282
1c	80 °C (2 min), 5 °C/min, 160 °C (1 min), 10 °C/min, 220 °C (8 min). Helium: 2 ml/min. Column: BGB173, 30 m x 0.25 mm ID, 0.25 µm.	1c: 13.143 min<br>(R)-2c: 22.512 min<br>(S)-2c: 22.583 mi
1d	80 °C (2 min), 5 °C/min, 160 °C (1 min), 10 °C/min, 220 °C (8 min). Helium: 2 ml/min. Column: BGB173, 30 m x 0.25 mm ID, 0.25 µm.	1d: 21.855 min<br>(+)-2d: 27.8 min<br>(-)-2d: 28.073 mi
2	80 °C (2 min), 5 °C/min, 160 °C (1 min), 10 °C/min, 220 °C (8 min). Helium: 2 ml/min. Column: BGB173, 30 m x 0.25 mm ID, 0.25 µm.	2: 5.342 min<br>(4R;6S)-4: 18.53 min<br>(4S;6R)-4: 18.62 min
3	80 °C (2 min), 5 °C/min, 160 °C (1 min), 10 °C/min, 220 °C (8 min). Helium: 2 ml/min. Column: BGB173, 30 m x 0.25 mm ID, 0.25 µm.	3: 7.305 or 7.492 min<br>P(-)-12a: 19.508 min<br>P(+)-12a: 19.073 min<br>D(-)-12b: 19.41 min<br>D(+)-12b: 19.635 mi
4a	110 °C (2 min); 10 °C/min, 118 °C;2 °C/min, 122 °C; 25 °C/min, 200 °C (1 min); 50 °C/min, 220 °C (4 min), Helium: 2 ml/min. Column: BGB175, 30 m x 0.25 mm ID, 0.25 µm.	4a: 9.1min<br>(S)-14a: 11.1 min<br>(R)-14a: 11.5 min
4b	80 °C (2 min); 2 °C/min, 220 °C (8 min), Helium: 2 ml/min. Column: BGB175, 30 m x 0.25 mm ID, 0.25 µm.	4b: 39.83 min<br>(S)-16a: 57.6 min<br>(R)-16a: 57.88 min
5a	80 °C (2 min); 2 °C/min, 220 °C (8 min), Helium: 2 ml/min. Column: BGB175, 30 m x 0.25 mm ID, 0.25 µm.	5a: 26.187 min<br>(S)-6a: 46.767 min<br>(R)-6a: 46.997mi
5b	80 °C (2 min); 2 °C/min, 220 °C (8 min), Helium: 2 ml/min. Column: BGB175, 30 m x 0.25 mm ID, 0.25 µm.	5b: 41.713 min<br>(S)-6b: 60.803 min<br>(R)-6b: 61.1 min
6	80 °C (2 min), 5 °C/min, 160 °C (1 min), 10 °C/min, 220 °C (8 min). Helium: 2 ml/min. Column: BGB173, 30 m x 0.25 mm ID, 0.25 µm.	6: 4.647 min<br>N(-)-8a: 14.233 min<br>N(+)-8a: 14.31 min<br>ABN(-)-8b: 13.133 min<br>ABN(+)-8b:
7	80 °C (2 min), 5 °C/min, 160 °C (1 min), 10 °C/min, 220 °C (8 min). Helium: 2 ml/min. Column: BGB173, 30 m x 0.25 mm ID, 0.25 µm.	8: 7.357 min<br>N(-)-10a: 16.37 min<br>N(+)-10a: 16.22 min<br>ABN(-)-10b: 15.28 min<br>ABN(+)-10b: 17.19 min

Baeyer-Viliiger oxidation of substrates

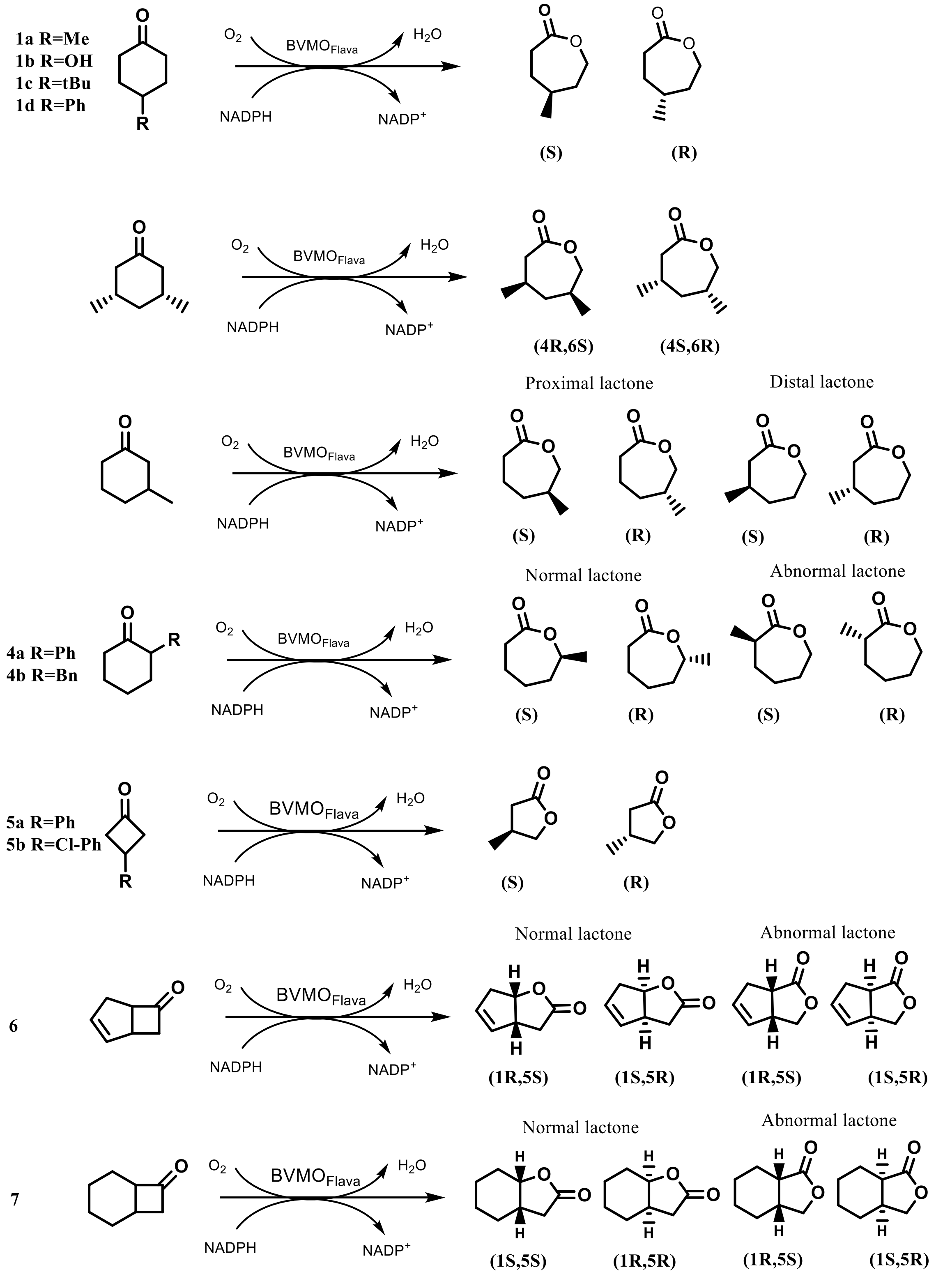

Figure 4 Baeyer–Villiger oxidation of a series of structurally diverse substrates catalyzed by $BVMO_{Flava}$.

References

[1] N. M. Kamerbeek, M. J. H. Moonen, J. G. M. van der Ven, W. J. H. van Berkel, M. W. Fraaije, D. B. Janssen, *Eur. J. Biochem.* **2001**, *268*, 2547-2557.

[2] K. Kostichka, S. M. Thomas, K. J. Gibson, V. Nagarajan, Q. Cheng, *J. Bacteriol.* **2001**, *183*, 6478-6486.

[3] R. S. Kantor, A. W. van Zyl, R. P. van Hille, B. C. Thomas, S. T. L. Harrison, J. F. Banfield, *Environ. Microbiol.* **2015**, *17*, 4929-4941.

[4] M. W. Fraaije, J. Wu, D. P. Heuts, E. W. van Hellemond, J. H. Spelberg, D. B. Janssen, *Appl. Microbiol. Biotechnol.* **2005**, *66*, 393-400.

[5] Y. C. J. Chen, O. P. Peoples, C. T. Walsh, *J. Bacteriol.* **1988**, *170*, 781-789.

[6] E. Romero, J. Ø. Rub, A. Mattevi, M. W. Fraaije, *Angew. Chem. Int. Ed.* **2016**, *55*, 15852-15855.

[7] K. Katoh, D. M. Standley, *Mol. Biol. Evol.* **2013**, *30*, 772-780.

[8] L. C. P. Goncalves, D. Kracher, S. Milker, M. J. Fink, F. Rudroff, R. Ludwig, A. S. Bommarius, *Adv. Synth. Catal.* **2017**, *359*, 2121-2131.
